# Resistive Oxygen Sensor Using Ceria-Zirconia Sensor Material and Ceria-Yttria Temperature Compensating Material for Lean-Burn Engine

**DOI:** 10.3390/s91108884

**Published:** 2009-11-05

**Authors:** Noriya Izu, Sayaka Nishizaki, Woosuck Shin, Toshio Itoh, Maiko Nishibori, Ichiro Matsubara

**Affiliations:** National Institute of Advanced Industrial Science and Technology (AIST), Advanced Manufacturing Research Institute, 2266-98 Anagahora, Shimo-Shidami, Moriyama-ku, Nagoya 463-8560, Japan; E-Mails: s-nishizaki@aist.go.jp (S.N.); w.shin@aist.go.jp (W.S.); itoh-toshio@aist.go.jp (T.I); m-nishibori@aist.go.jp (M.N.); matsubara-i@aist.go.jp (I.M.)

**Keywords:** air-to-fuel ratio, automobile, gas sensor, solid electrolyte, resistive-type

## Abstract

Temperature compensating materials were investigated for a resistive oxygen sensor using Ce_0.9_Zr_0.1_O_2_ as a sensor material for lean-burn engines. The temperature dependence of a temperature compensating material should be the same as the sensor material; therefore, the Y concentration in CeO_2_-Y_2_O_3_ was optimized. The resistance of Ce_0.5_Y_0.5_O_2-δ_ was independent of the air-to-fuel ratio (oxygen partial pressure), so that it was confirmed to function as a temperature compensating material. Sensor elements comprised of Ce_0.9_Zr_0.1_O_2_ and Ce_0.5_Y_0.5_O_2-δ_ were fabricated and the output was determined to be approximately independent of the temperature in the wide range from 773 to 1,073 K.

## 1. Introduction

Resistive oxygen sensors have recently attracted much attention due to their simple structure [1-5]. We have studied resistive oxygen sensors using cerium oxide as a sensor material [6-10], which has advantageous features such as durability against corrosive gases in vehicle exhaust [11-13]. The response time of a sensor using a cerium oxide thick film was improved by reducing the particle size from 2,000 to 100 nm in the thick film [14]. However, resistive oxygen sensors using n-type oxide semiconductors not only have resistance that is dependent on oxygen partial pressure, but also on temperature, and a large temperature dependence is a problem for a sensor. Generally, temperature compensating materials are used with a sensor material to solve such a problem [15-22]. Solid electrolytes have been previously suggested for use as temperature compensating materials [8,23].

A temperature compensating material was reported for lambda sensors [24], which can detect fuel-rich or fuel-lean air-to-fuel ratios for improvement of the conversion ratio of three-way catalysts. This report [24] suggested that yttria-doped zirconia should be used as a temperature compensating material for lambda sensors using a ceria-zirconia thick film as a sensor material.

For improvement in the mileage of vehicles, lean-burn engines have been proposed and recently commercialized [5]. We previously reported [23] the possibility of applying temperature compensating materials for lean-burn engines. In this report, we have made a detailed investigation of temperature compensating materials used for resistive oxygen sensors of Ce_0.9_Zr_0.1_O_2_ (CeZr10), which have a fast response and low resistance [14], in a lean-burn engine. First, temperature compensating materials suitable for CeZr10 were considered. The temperature dependence of the resistance for CeZr10 is large, as described in detail in Section 3, so that a solid electrolyte with a large temperature dependence of resistance is required. In the case of lean-burn, ceria system solid electrolytes are suitable, due to the high oxygen partial pressure, although this system has large electronic conductivity in low oxygen partial pressure [25]. Because the sensor material is also a ceria system, ceria solid electrolytes are used as the temperature compensating material for the oxygen sensor in a lean-burn engine. The temperature dependence of the resistance for yttria-doped ceria is larger than that for ceria doped with Sm, La, or Ca [26]. It was reported that the temperature dependence of resistance increased with increasing Y concentration in the CeO_2_-Y_2_O_3_ system [21]. Therefore, the Y concentration was optimized to obtain a suitable temperature compensating material for CeZr10. Furthermore, sensor elements comprising the sensor and temperature compensating materials were fabricated and the temperature dependent output of the sensor element was investigated.

## 2. Experimental

### 2.1. Sample preparation

#### 2.1.1. Ceria-yttria powders

Solutions of Ce(NO_3_)_3_·6H_2_O and Y(NO_3_)_3_·6.2H_2_O were prepared using distilled water and stirred for several minutes. The concentrations of Ce^3+^ or Y^3+^ ([Ce^3+^] or [Y^4+^]) were 0.1 mol/dm^3^. The Ce(NO_3_)_3_ and Y(NO_3_)_3_ solutions were then mixed in ratios of [Y^3+^]/([Y^3+^]+[Ce^3+^]) = 0.20, 0.30, 0.40, 0.50, 0.60 and 0.70, which were denoted as CeY20, CeY30, CeY40, CeY50, CeY60, and CeY70, respectively. The mixed solution was mixed with aqueous ammonia and the resulting precipitate was then filtrated to obtain a white gel. The white gel was mixed with commercially available carbon powder using a hybrid mixer (Keyence Corporation, HM-500). The mixture was dried at 343 K in air for several hours and then calcined at 1,173 K to obtain a fine yttria-doped ceria powder.

#### 2.1.2. Ceria-zirconia powder

Zirconia-doped ceria powder with [Zr^4+^]/([Zr^4+^] + [Ce^3+^]) = 0.10 (CeZr10) was prepared using the same method as that for the CeY20-CeY50 powders using ZrO(NO_3_)_2_ solution instead of Y(NO_3_)_3_ solution.

#### 2.1.3. Thick films

A paste was first prepared by mixing the fine yttria-doped ceria or zirconia-doped ceria powder with an organic binder that included terpineol and ethyl cellulose. The paste was screen-printed on Al_2_O_3_ substrates. The screen printed thick films were calcined at 773 K for 5 h in air, and then sintered at 1,373 K. The thicknesses of the thick films were in the range from 6 to 8 μm.

#### 2.1.4. Electrodes

Pt was used as the electrode material. The Pt electrodes had an interdigital structure. Three different electrode patterns were used in this study, the details of which are described in other sections.

### 2.2. Evaluation

The thick films were characterized using X-ray diffraction, (XRD; Cu Kα radiation, (RINT2100V/PC, Rigaku Corporation) and scanning electron microscopy, (SEM; JSM-6335F, Jeol).

For optimization of the Y concentration in the CeO_2_-Y_2_O_3_ system, the following experiment was carried out: sensor elements using the electrode patterns reported in [23] were placed in a furnace and heated in the range from 773 to 973 K. The resistance of the thick film was measured in a flow of 18.1%O_2_ in N_2_ or 0.20%O_2_ in N_2_. The activation energy of resistance, i.e., the temperature dependence of resistance, was calculated from an Arrhenius plot of reciprocal temperature versus the logarithm of resistance.

An apparatus for evaluation of the sensors in a model exhaust gas was used to obtain the resistance of the sensor material or the temperature compensating material, or the output of the sensor elements. The details of the apparatus are as follows:

The gas obtained after combustion of a gas mixture of propane, oxygen, and nitrogen over a combustion catalyst was used as the model gas to simulate exhaust gas. The resistance of the thick films and the output of the sensors were measured in the model gas. The details of the experimental method have been reported in [27].

Excess oxygen factor λ was defined as follows:

(1) $$\lambda =\frac{\left\{C({\text{O}}_{2})/C({\text{C}}_{3}{\text{H}}_{8})\right\}}{{\left\{C({\text{O}}_{2})/C({\text{C}}_{3}{\text{H}}_{8})\right\}}_{\text{stoich}}},$$

where *C*(O_2_) and *C*(C_3_H_8_) are the concentrations of oxygen and propane, respectively, and {C(O_2_)/C(C_3_H_8_)}_stoich_ is the stoichiometric ratio. The general excess air factor λ_air_ is described as follows:

(2) $$\lambda_{\text{air}}=\frac{\left\{w(\text{air})/w(\text{fuel})\right\}}{{\left\{w(\text{air})/w(\text{fuel})\right\}}_{\text{stoich}}}$$

where *w*(air) and *w*(fuel) are the weight of air and fuel, respectively, and {*w*(air)/*w*(fuel)}_stoich_ is the stoichiometric air-to-fuel ratio. Therefore, if the value of λ is the same as that of λ_air_, the oxygen partial pressures are significantly different. In the case of the same oxygen partial pressure, the relationship between λ and λ_air_ is expressed as:

(3) $$\lambda_{\text{air}}\approx 0.15\times \lambda +0.87(1.1\leq \lambda \leq 1.6)$$

Thus, it was concluded that the results in this study were measured near λ_air_ = 1.

The oxygen partial pressure was measured by a zirconia sensor maintained at 1,008 K as a standard, i.e., the temperature of the measured gas was 1,008 K. The oxygen partial pressure changed sharply at λ = 1. The oxygen partial pressures at λ > 1 and λ < 1 were high and low, respectively. The regions of λ > 1 and λ < 1 are referred to as lean and rich, respectively.

**Figure 1.**
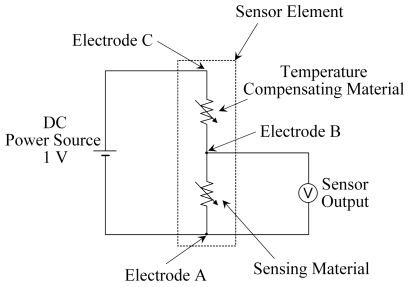
Electrical circuit for the sensor element using the temperature compensating material.

The electrical circuit for the sensor element using the temperature compensating material is shown in Figure 1. A power source with a constant voltage of 1 V was connected in series to the sensor material and the temperature compensating material, and the voltage difference of the sensor material (voltage between electrodes AB) was measured as the output of the sensor element using the temperature compensating material.

## 3. Results and Discussion

### 3.1. Optimization of Y concentration

**Figure 2.**
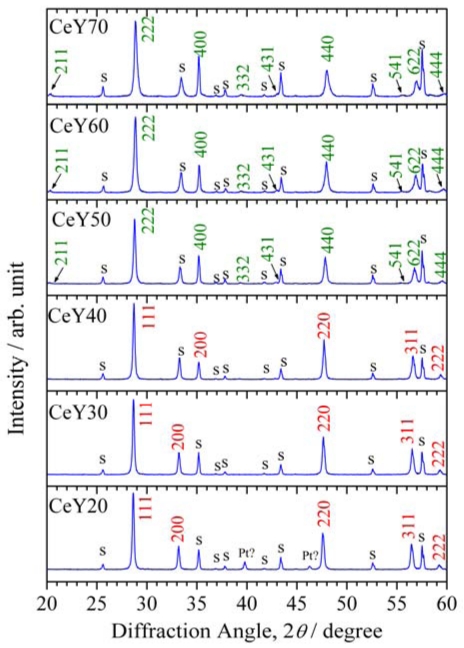
XRD patterns for thick films with various Y concentrations, fired at 1,373 K. “CeY*x*” indicates a composition of Ce_1-_*_x_*_/100_Y*_x_*_/100_O_2-δ_. “s” indicates the peak from the substrate.

XRD patterns for the thick films fired at 1,373 K are shown in Figure 2. XRD analysis was carried out for thick films on alumina substrates. In the case of CeY20, the analysis was carried out for thick films with Pt electrodes, while for the samples with other concentrations of Y, thick films specially prepared for XRD analysis were used without Pt electrodes.

All of the XRD peaks in patterns for CeY20 to CeY40 were assigned to a fluorite structure, excluding those peaks from the alumina substrate, but some peaks in the patterns for CeY50 to CeY70 were not assigned to a fluorite structure. Y_2_O_3_ has a C-rare earth structure; therefore, the crystal structure was considered to change from fluorite type to the C-rare earth type structure. All of the XRD peaks in the patterns for CeY50 to CeY70 were assigned to the C-rare earth structure.

The angle of the peaks from the thick films increased with increasing Y concentration, which indicates the lattice constant decreased with increasing Y concentration. The coordination numbers of the cations in the fluorite and C-rare earth structures are 8 and 6, respectively. The ionic radius of Ce^4+^ with a coordination number of 6 or 8 is 0.087 or 0.097 nm, respectively, while that of Y^3+^ with a coordination number of 6 or 8 is 0.0900 or 0.1019 nm, respectively [28]. Therefore, the ionic radius of Y^3+^ with a coordination number of 6 is smaller than that of Ce^4+^ with a coordination number of 8, which may be the reason for the decrease in the lattice constant with increasing Y concentration.

**Figure 3.**
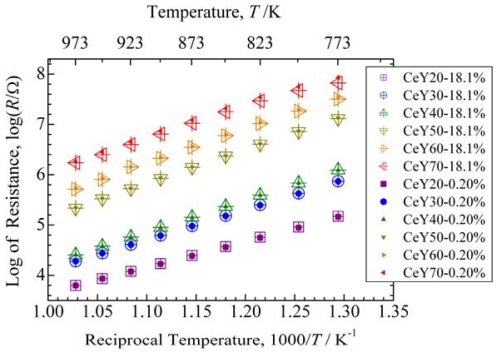
Resistance of thick films with various Y concentrations in the range from 773 to 973 K. “CeYx” indicates a composition of Ce_1-_*_x_*_/100_Y*_x_*_/100_O_2-δ_. The values after the composition indicate oxygen concentration.

McCullough and Britton proposed a complete solid solution for the CeO_2_-Y_2_O_3_ system and a continuous structural transition between CeO_2_ and Y_2_O_3_ [29]. However, Longo and Podda reported the existence of a two phase region [30]. Djurovic *et al.* summarized several phase diagrams of the CeO_2_-Y_2_O_3_ system reported so far [31]. In this study, it was not concluded that the medium region in the CeO_2_-Y_2_O_3_ system has a complete solid solution or two phase region, because the XRD pattern of the fluorite structure was similar to that for the C-rare earth structure, and the difference in the lattice constant of the two structures was only slight. We do not discuss this phenomenon in detail, because it is not within the scope of interest. However, it became clear that no third phase with a different structure was present in the samples. Figure 3 shows the resistance of thick films with various Y concentrations in the range from 773 to 973 K.

The resistance increased monotonically with increasing Y concentration. The gap of the resistance between CeY40 and CeY50 was larger than that for the other samples, which may be attributable to the difference of the crystal structures.

The resistances in an oxygen concentration of 18.1% were the same as that for 0.20% in the case of CeY20 to CeY50. For CeY60, the resistances at lower temperatures were different for oxygen concentrations between 18.1% and 0.20%. For CeY70, the resistances in the temperature range from 773 to 973 K were different for oxygen concentrations between 18.1% and 0.20%. The independence of the resistance with respect to the oxygen concentration is attributable to oxide ion conduction [32,33] for CeY20 to CeY50. The difference in the resistance between two oxygen partial pressures could be attributable to electrical conduction in the case of CeY60 and CeY70.

**Figure 4.**
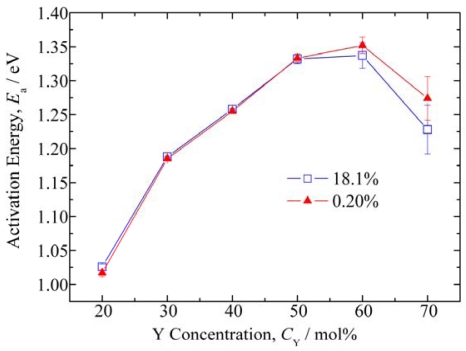
Dependence of activation energy on the Y concentration, calculated from the data shown in Figure 3.

Figure 4 shows the activation energies calculated from the data shown in Figure 3. The activation energy increased with increasing Y concentration from CeY20 to CeY60, but decreased from CeY60 to CeY70. For the samples of more than 50 mol% Y, major cations are not Ce but Y. This means that the secondary element is Ce for the samples of more than 50 mol% Y. Thus, Ce may be the origin of scattering for oxide ion flow in the high concentrations of Y. Ce concentration decreases with increasing Y concentrations. This may be the reason for the decreasing activation energy in the high concentrations of Y. For CeY60 and CeY70, the activation energy was different when the oxygen concentration changed, which indicates that these materials are unsuitable as temperature compensating materials.

Measurement of the temperature dependence of resistance for CeZr10 using the same electrode structure as the samples in Figures 3 and 4 gave activation energies of 1.52 and 1.50 eV in oxygen concentrations of 18.1% and 0.20%, respectively. Therefore, it was concluded that CeY50 is the most suitable temperature compensating material of those examined in this study with respect to activation energy and the independence of resistance to oxygen concentration.

### 3.2. Sensor element

**Figure 5.**
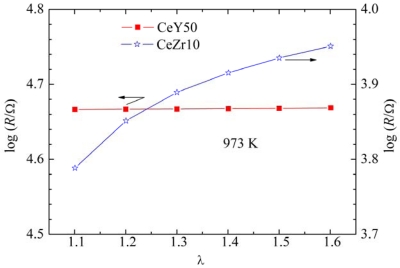
Dependence of the resistance on λ for CeY50 (Ce_0.5_Y_0.5_O_2-δ_) and CeZr10 (Ce_0.9_Zr_0.1_O_2_).

Figure 5 shows the dependence of resistance on λ for CeY50 and CeZr10. In this measurement, the electrodes for CeY50 and CeZr10 were the same and had an interdigital structure with a finger width of 0.1 mm, a finger length of 3.2 mm, a finger space of 0.2 mm, and a finger pair number of 7. The resistance of CeY50 was independent of λ, which satisfies the required property for a temperature compensating material. The resistance of CeZr10 increased with increasing λ.

**Figure 6.**
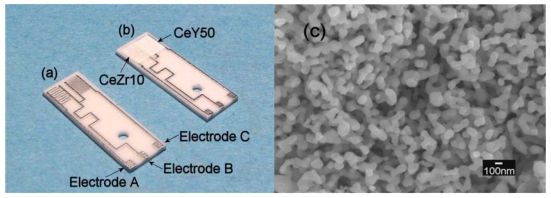
Fabricated sensor element. (a) Electrode patterns for the sensor material and the temperature compensating material on one substrate with a size of 15 × 5 mm^2^. (b) Overview of the sensor element, comprised of thick films of the sensor material and the temperature compensating material. (c) SEM image of the CeY50 (Ce_0.5_Y_0.5_O_2-δ_) sample surface.

Figure 6(a) shows electrode patterns for the sensor material and the temperature compensating material on one substrate with a size of 15 × 5 mm^2^. The electrodes for the sensor materials are shown at the lower left of Figure 6(a) and those for the temperature compensating material are shown at the upper right of Figure 6(a).

The resistance of the temperature compensating material was larger than that of the sensor material; therefore, the electrodes for the former had a smaller finger space and a larger finger pair number than those of the latter. Optimization resulted in similar resistances. Figure 6(b) shows a sensor element comprised of thick films of the sensor material and the temperature compensating material. Figure 6(c) shows an SEM image of the CeY50 sample surface. The thick film has a porous structure and its particle size and thickness were approximately 130 nm and 7.5 μm, respectively.

**Figure 7.**
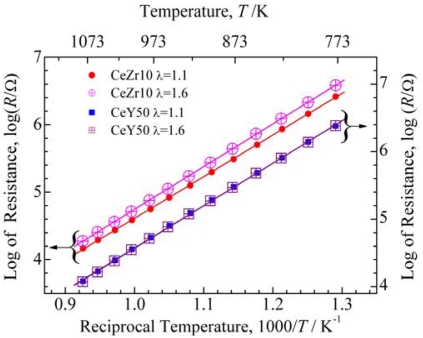
Resistance of the temperature compensating material (CeY50:Ce_0.5_Y_0.5_O_2-δ_) and the sensor material (CeZr10:Ce_0.9_Zr_0.1_O_2_) on the sensor element shown in Figure 5(b).

The resistance of the temperature compensating material and the sensor material of the sensor element (Figure 6(b)) are shown in Figure 7. Both the resistance of the temperature compensating material and the sensor material were the same, so that two vertical axes have been used in Figure 7 for clarity. The resistance of the temperature compensating material was independent of λ but that of the sensor material was dependent on λ; therefore, it was confirmed that both of the materials functioned as expected.

The activation energy for the resistance of CeY50 was 1.25 eV in both case of λ = 1.1 and 1.6, while the activation energies of CeZr10 were 1.23 and 1.26 eV in the case of λ = 1.1 and 1.6, respectively. Therefore, it was confirmed that the temperature dependency of the resistance for CeY50 and CeZr10 were almost the same. These activation energies were slightly smaller than that shown in Figure 4, which may be due to the difference in the electrode structures.

**Figure 8.**
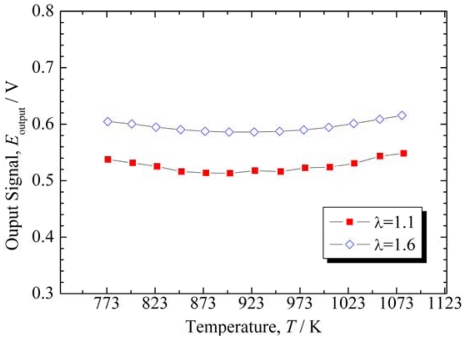
Output signal of the sensor element obtained using the electric circuit shown in Figure 1.

Figure 8 shows the output signal of the sensor element obtained using the electric circuit shown in Figure 1. For λ = 1.1 and 1.6, the output was slightly dependent on the temperature, as indicated by the slightly concave curves shown in Figure 8. The maximum, medium, and minimum outputs were 0.548, 0.531, and 0.513 V at λ = 1.1, and 0.615, 0.601, and 0.586 V at λ = 1.6, respectively. The difference between the medium values of λ = 1.6 and 1.1 (Δ*E*_output_) is 0.07 V, so that Δ*E*_output_/Δλ = 0.14 is obtained. The output fluctuated from the medium values with a range of 0.018 V or 0.015 V in the case of λ = 1.1 and 1.6, respectively.

The fluctuations represent 0.13 (=0.018/(Δ*E*_output_/Δλ)) in λ, so that the sensor element can detect a difference of 0.26 in λ. When λ_air_ is calculated from λ using Equation (3), a difference of 0.26 in λ indicates a difference of 0.04 in λ_air_.

The power source voltage (Figure 1) was set to 1 V for the results in Figure 8. When the power source voltage was set higher, the sensor element output responded as the resistance of the temperature compensating material became higher. This may be attributed to an increase in the interface resistance between the materials and the electrode, because as the power source voltage increases, the current density increases. In the case of the temperature compensating material, the charge carriers are oxide ions, so that the reaction in Equation (4) occurs at the electrode:

(4) $$1/2{\text{O}}_{2}+2{\text{e}}^{-}\to {\text{O}}^{2-}$$

When this reaction is much faster, the interface resistance is not apparent. However, if there is an increase in electrons supplied from the electrodes, then Equation (4) becomes a rate-limiting step due to the relatively slow kinetics of the reaction. This may be the reason for the increase in interface resistance. Therefore, it is necessary to ensure that the power source voltage is not too large.

## 4. Conclusions

Temperature compensating materials were investigated for a resistive oxygen sensor using CeZr10 (Ce_0.9_Zr_0.1_O_2_) as a sensor material for lean-burn engines. The temperature compensating material should have the same temperature dependence of resistance as the sensor material and resistance that is independent of oxygen partial pressure. As a result of Y concentration optimization in the CeO_2_-Y_2_O_3_ material for the temperature compensating material, it was revealed that CeY50 (Ce_0.5_Y_0.5_O_2-δ_) had the same activation energy of resistance as CeZr10 and resistance that was independent of λ. For this composition, XRD analysis revealed no other phase except for fluorite or C-rare earth structures. Therefore, CeY50 was confirmed to function as a suitable temperature compensating material. Sensor elements comprised of CeZr10 and CeY50 as sensor and temperature compensating materials (Figure 6(b)) were fabricated, and the dependence of the sensor element output on temperature was investigated. The electrodes in the sensor element were optimized to adjust the resistance of CeZr10 to that of CeY50. The output was approximately independent of temperature in a wide range from 773 to 1,073 K and detection limits were clarified; the sensor could distinguish a difference of 0.26 and 0.04 in λ and λ_air_, respectively.
